# Barriers to Routine Gynecological Care in Young Adult Females in the United States

**DOI:** 10.1089/whr.2025.0015

**Published:** 2025-05-19

**Authors:** Anne M. Clark, Mireya C. Long, Brianna M. Magnusson

**Affiliations:** ^1^ Neuroscience Center, College of Life Sciences, Brigham Young University, Provo, Utah, USA.; ^2^ Department of Public Health, College of Life Sciences, Brigham Young University, Provo, Utah, USA.

**Keywords:** women’s health services, preventative health services, well-woman visit, patient–provider relations

## Abstract

**Introduction:**

Well-woman exams (WWEs) are important preventive health care; however, many do not regularly receive these exams. Understanding barriers to timely care is important in improving health care delivery and health outcomes.

**Methods:**

We conducted a panel survey of 1000 U.S. females 18–30 years of age. Barriers to gynecological care were assessed in four domains: financial, practical, procedural, and health care provider related. Differences between groups were assessed using *t*-test and analysis of variance, and logistic regression was used to examine the association between barriers and delayed care.

**Results:**

Respondents were on an average 24.5 years old and the majority were White, non-Hispanic (63%). Respondents were categorized by WWE screening status: never screened (24%), delayed screening (>1 year) (30%), and on-time screening (46%). Those who had delayed WWE expressed higher practical, procedural, and provider barriers compared with those with on-time exams. After adjusting for other barrier types and sociodemographic characteristics, lack of insurance was associated with more than twice the odds of delayed WWE (odds ratio [OR]: 2.61 95% confidence interval [CI]: 1.41–4.92) and a one-point increase in the provider barriers mean scale was associated with nearly 60% increased odds (OR: 1.59; 95% CI: 1.16–2.17) of having delayed WWE.

**Conclusions:**

The patient experience with the health care provider, along with insurance coverage, is significantly associated with delayed WWE. These results indicate that in the presence of insurance coverage, providers have a significant role in creating an environment that supports the timeliness of WWE in young adult patients.

## Introduction

Well-woman exams (WWEs) are important preventive health care for females. WWEs screen for common infections, health conditions, and gynecological cancers, and are intended to be performed on healthy and asymptomatic patients.^1^ Additionally, regular preventive screenings allow health care providers to consult with patients about sexually transmitted infection (STI) testing, fertility, sexual health and dysfunction, immunizations, preventive screenings (*e.g.,* blood pressure, diabetes), and maintaining a healthy lifestyle through nutrition, physical activity, and weight management.^2,3^

This study explored barriers to preventive gynecological care in U.S. females aged 18–30 years of age. The early adult years are a period of sexual exploration for many that increases the need for contraception and the risk of STIs. Further, this period encompasses the average age of first birth for U.S. females (26.3 years) and represents a period when fertility care and preconception planning may be warranted.^4^ Although young adults are not typically at high risk for cancer, cancer rates in persons <50 years old are increasing.^5^ One study reported a 3.39% annual increase in late-stage cervical cancer incidence in females aged 30–34 years.^6^ Cervical cancer has a 91% 5-year survival rate when localized, but survival declines to 19% in distant stages underscoring the importance of routine cancer screening.^7^ Young adults may also experience more financial barriers relative to other groups as they are more likely to be actively pursuing education and less likely to have stable sources of income. Finally, this young adult period can be a particularly salient period for establishing healthy habits (*e.g.,* good nutrition, physical activity, avoidance of substances, blood pressure monitoring, and diabetes screening) that include preventive gynecological screenings.^8^

### Aims and hypotheses

This study aims to (i) describe barriers to routine gynecological care in young adult U.S. females, (ii) examine the association between specific types of barriers (*e.g.,* financial, practical, procedural, and provider experience barriers) and routine gynecological care, and (iii) explore whether the barriers to routine gynecological care differ across sociodemographic variables.

## Materials and Methods

### Sample

A survey of 1000 females 18–30 years old in the United States was administered in December 2022. Respondents were recruited through Qualtrics (Provo, UT, USA), a worldwide software company that operates a proprietary panel and recruits through social media, web publishers, and global partners. Panelists who met the initial screening criteria (18–30 years of age, female sex at birth) received an email invitation to participate. The invitation included a link to the survey as well as information about the survey length, topics of inquiry, and the value of compensation (<$5 in reward points credited to their Qualtrics account). Those who followed the survey link were taken to a consent page informing them of their rights as a research participant, including the ability to skip questions or withdraw at any time. Respondents indicated consent by selecting “I agree to participate.” After entering the survey, respondents were further screened for eligibility (presence of reproductive organs) and then proceeded with the survey. To increase the representativeness of the sample, sampling quotas based on the 2020 U.S. Census for region of the United States, race and ethnicity, low education, and low income were used.^9^ The study was approved by the Human Research Protection Program at Brigham Young University. Eligible persons were 18–30 years of age, female sex, any gender, with a uterus and ovaries and currently resided in the United States.

### Measurement

The survey lasted approximately 15 minutes. Items included financial, practical, procedural, and health care provider experience barriers to gynecological care along with, nongynecological health care seeking behaviors, sexual and reproductive health history, and demographic characteristics.

Gynecological care history was assessed using 24 questions adapted from the 2017–2019 National Survey of Family Growth (NSFG).^10^ Questions included whether they had ever seen a women’s health care provider for any reason, had ever received a pap smear, pelvic exam, and/or breast exam from any provider, age at first WWE, approximate number of visits in the last year and the reason for their most recent visit to a women’s health care provider. Participants were classified into three groups based on ever having a WWE and the timing of that exam: (i) never had a WWE, (ii) most recent WWE >1 year ago (delayed), and (iii) most recent WWE within the last year (on-time).

Questions related to financial barriers were adapted from the Behavioral Risk Factor Surveillance System (BRFSS) and the National Health Interview Survey (NHIS).^11,12^ All respondents indicated if they had health insurance. Insured respondents were asked about the perceived amount of any co-pay for receiving preventive care and their ability to cover that co-pay. Uninsured respondents answered similar questions assessing the perceived amount of and ability to pay out-of-pocket costs for preventive care. Additionally, respondents were asked if they had skipped or delayed care due to cost concerns or had difficulty paying for medical expenses in the previous year.

Perceived access to gynecological care was assessed with six author-created statements (see Supplementary Table S1). Questions focused on the practical aspects of access such as finding a provider, making an appointment, and finding transportation (practical barriers). Barriers associated with the procedural aspects of gynecological care were evaluated using seven author-created statements (see Supplementary Table S2). Items focused on procedural aspects such as being undressed and speculum insertion. Barriers associated with the provider experience were assessed using 21 author-created statements. Items focused on the patient–provider interaction including questions about feeling judged by the provider, the provider listening to the patient, and the provider explaining the purpose of tests and procedures. For all barriers scales respondents rated agreement on a 5-point Likert scale from 1 (strongly agree) to 5 (strongly disagree).

**Table 1.** Demographic Characteristic of a Sample of Young Adult U.S. Females, Total Sample and by Timing of Well-Woman Exam

	Total sample *n* = 1000	Never *n* = 237	Delayed *n* = 297	On-time *n* = 462	
Variable	*n* (%)	*n* (%)	*n* (%)	*n* (%)	*p*
Age (mean, SD)	24.5 (3.61)	22.19 (3.28)	25.20 (3.34)	25.16 (3.45)	<0.001
Gender identity (*n* = 998)					
Cisgender female	906 (90.78)	210 (88.61)	268 (90.24)	424 (92.17)	0.287
Something else	92 (9.22)	27 (11.39)	29 (9.76)	36 (7.83)	
Marital status (*n* = 1000)					
Married	213 (21.30)	26 (10.97)	62 (20.88)	124 (26.84)	<0.001
Cohabitating	279 (27.90)	45 (18.99)	95 (31.99)	138 (29.87)	
Never married	486 (48.60)	161 (67.93)	130 (43.77)	193 (41.77)	
Divorced/widowed/separated	22 (2.20)	5 (2.11)	10 (3.37)	7 (1.52)	
Race/ethnicity (*n* = 1000)					
White, non-Hispanic	634 (63.40)	133 (56.12)	203 (68.35)	298 (64.50)	0.016
Hispanic	160 (16.0)	51 (21.52)	49 (16.50)	59 (12.77)	
Black, non-Hispanic	125 (12.50)	28 (11.81)	30 (10.10)	66 (14.29)	
Multiracial	39 (3.90)	12 (5.06)	7 (2.36)	18 (3.90)	
Other, single race	42 (4.20)	13 (5.49)	8 (2.69)	21 (4.55)	
Current student (*n* = 1000)					
Yes	318 (31.80)	106 (44.73)	75 (25.25)	137 (29.65)	<0.001
No	682 (68.20)	131 (55.27)	222 (74.75)	325 (70.35)	
Education (*n* = 1000)					
Current high school student, completed high school, or less than high school	340 (34.0)	112 (47.26)	90 (30.30)	135 (29.22)	<0.001
Current or completed associates/trade/technical/vocational/occupational	149 (14.9)	30 (12.66)	41 (13.80)	78 (16.88)	
Current university student or completed some college (no degree)	241 (24.1)	65 (27.43)	76 (25.59)	99 (21.43)	
Completed bachelor’s degree or higher	270 (27)	30 (12.66)	90 (30.30)	150 (32.47)	
Household income (*n* = 1000)					
<$24,999	246 (24.60)	79 (33.33)	69 (23.23)	96 (20.78)	<0.001
$25,000−$44,999	252 (25.20)	63 (26.58)	77 (25.93)	112 (24.24)	
$45,000−$64,999	186 (18.60)	32 (13.50)	69 (23.23)	83 (17.97)	
$65,000−$94,999	175 (17.50)	32 (13.50)	52 (17.51)	91 (19.70)	
$>$95,999	141 (14.10)	31 (13.08)	30 (10.10)	80 (17.32)	
Number of people supported by income (*n* = 1000)					
1 person (they support no one other than themselves)	316 (31.60)	92 (38.82)	101 (34.01)	122 (26.41)	0.018
2 people	248 (24.80)	47 (19.83)	83 (27.95)	118 (25.54)	
3 people	181 (18.10)	35 (14.77)	47 (15.82)	98 (21.21)	
4 people	160 (16.00)	40 (16.88)	78 (16.88)	42 (14.14)	
5 or more people	95 (9.50)	23 (9.70)	24 (8.08)	46 (9.96)	
Primary health care provider (*n* = 1000)					
Yes	810 (81.00)	164 (69.20)	229 (77.10)	413 (89.39)	<0.001
No	190 (19.00)	73 (30.80)	68 (22.90)	49 (10.61)	
Time since last routine checkup (*n* = 1000)					
<1 year	696 (69.60)	131 (55.27)	144 (48.48)	417 (90.26)	<0.001
1–2 years	159 (15.90)	48 (20.25)	84 (28.28)	27 (5.84)	
2 or more years	145 (14.50)	58 (24.47)	69 (23.23)	18 (3.90)	
Average number of doctors’ visits per year (*n* = 999)					
0–1 visits	274 (27.43)	103 (43.46)	94 (31.65)	77 (16.70)	<0.001
2–3 visits	454 (45.45)	91 (38.40)	132 (44.44)	228 (49.46)	
4–5 visits	171 (17.12)	28 (11.81)	45 (15.15)	97 (21.04)	
6 or more visits	100 (10.01)	15 (6.33)	26 (8.75)	59 (12.80)	
Insurance coverage (*n* = 990)					
Yes	884 (89.29)	184 (78.30)	258 (88.05)	438 (95.63)	<0.001
No	106 (10.71)	51 (21.70)	35 (11.95)	20 (4.37)	
Did not get needed medical care in the last 12 months due to cost concerns (*n* = 980)					
Yes	366 (37.20)	98 (41.88)	123 (41.98)	145 (32.01)	<0.01
No	618 (62.80)	136 (58.12)	170 (58.02)	308 (67.99)	
Difficulty paying for medical expenses in the last year (*n* = 979)					
Yes	285 (28.99)	50 (21.46)	101 (34.47)	134 (29.58)	<0.01
No	698 (71.01)	183 (78.54)	192 (65.53)	319 (70.42)	

SD, standard deviation.

Those who had not had a WWE in the last year were asked 21 author-created questions regarding reasons they may not have had a WWE (Supplementary Table S3). Additionally, the subset of participants who reported that they had ever intentionally delayed recommended pap smear testing or recommended STI testing were asked a series of six (pap smear; Supplementary Table S4) or seven (STI testing; Supplementary Table S5) author-created questions about the reasons they may have chosen to delay those exams. For all three sets of questions, respondents indicated agreement on a 5-point Likert scale (1 = strongly agree to 5 = strongly disagree).

All author-created items were informed by an evaluation of the literature on health care barriers, experiences with gynecological care, and patient−provider experiences.^13–15^ Questions were pilot-tested among a convenience sample of students in the demographic of interest for clarity and performance and modified as needed to arrive at the administered questions. Additionally, Qualtrics administered the survey to a sample of 50 eligible respondents, and the data were reviewed for performance.

To assess whether there were additional barriers to gynecological care compared with other types of health care we asked three questions modeled after questions from BRFSS and NHIS.^11,12^ Questions included whether they had a primary care provider, time since last checkup, and average number of doctor visits per year.

Sexual history questions included whether the respondent had ever had a sexual experience with another person, age at first sexual experience, and number of sexual partners in the past year. We defined a sexual experience as when “one person uses any body part or object to stimulate the genitals of another person.” Reproductive history questions included age at menarche, whether the respondent had a menstrual period in the last 6 months, perceived normality of menstrual cycle length and bleeding patterns, and pregnancy history.

Respondents reported age in years, biological sex, gender, sexual orientation, marital status, race, ethnicity, education, income, and household size. Gender response options included: cisgender woman, cisgender man, genderqueer/nonbinary, or gender fluid, transgender woman, transgender man, or a gender not listed here. Only 33 people chose genderqueer/nonbinary, or gender fluid, and an additional 58 persons selected a gender not listed. None of the participants reported being transgender. For analysis, these categories were collapsed into cisgender females versus nonfemale gender identity. Sexual orientation response options included: heterosexual or straight, gay or lesbian, bisexual, asexual, pansexual, queer, a sexual orientation not listed here, or I prefer not to say. For analysis, we collapsed categories with small cell counts yielding a 4-level variable (heterosexual, bisexual, gay/lesbian, something else). Other demographic variables were operationalized as indicated in Table 1.

**Table 2.** Sexual and Reproductive Health Characteristics for a Sample of Young Adult U.S. Females, Total Sample and by Timing of Well-Woman Exam

	Total sample *n* = 1000	Never *n* = 237	Delayed *n* = 297	On-time *n* = 462	
Variable	*n* (%)	*n* (%)	*n* (%)	*n* (%)	*p*
Sexual orientation (*n* = 996)					
Heterosexual	722 (72.20)	154 (64.98)	201 (67.68)	364 (78.79)	<0.001
Bisexual	166 (16.60)	38 (16.03)	56 (18.86)	71 (15.37)	
Gay/lesbian	42 (4.20)	13 (5.49)	17 (5.72)	12 (2.60)	
Something else	70 (7.0)	32 (13.50)	23 (7.74)	15 (3.25)	
Age at first menstrual period (*n* = 994)					
10 years old or younger	155 (15.53)	29 (12.24)	43 (14.58)	83 (17.97)	0.244
11 years old	173 (17.33)	42 (17.72)	53 (17.97)	75 (16.23)	
12 years old	297 (29.76)	83 (35.02)	89 (30.17)	125 (27.06)	
13 years old	200 (20.04)	51 (21.52)	57 (19.32)	92 (19.91)	
14 years old or older	173 (17.33)	32 (13.50)	53 (17.97)	87 (18.83)	
Have you had at least one period in the last 6 months? (*n* = 994)					
Yes	898 (89.98)	222 (94.07)	275 (92.59)	397 (86.12)	<0.001
No	100 (10.02)	14 (5.93)	22 (7.41)	64 (13.88)	
Would you consider your menstrual cycle length relatively normal? (*n* = 994)					
Yes	730 (73.15)	172 (72.88)	217 (73.06)	338 (73.32)	0.992
No	268 (26.85)	64 (27.12)	80 (26.94)	123 (26.68)	
Would you consider your bleeding length relatively normal? (*n* = 994)					
Yes	764 (76.55)	188 (79.66)	224 (75.42)	350 (75.92)	0.453
No	234 (23.45)	48 (20.34)	73 (24.58)	111 (24.08)	
Ever had a sexual experience with another person (*n* = 996)					
Yes	852 (85.20)	163 (68.78)	258 (86.87)	427 (92.42)	<0.001
No	148 (14.80)	74 (31.22)	39 (13.13)	35 (7.58)	
Age at first sexual experience with another person (*n* = 848)					
<12 years	40 (4.69)	8 (4.91)	21 (8.14)	11 (2.58)	0.085
12–13 years	66 (7.75)	15 (9.20)	16 (6.20)	34 (7.96)	
14–15 years	205 (24.06)	43 (26.38)	56 (21.71)	106 (24.82)	
16–17 years	262 (30.75)	49 (30.06)	81 (31.40)	129 (30.21)	
18+ years	279 (32.75)	48 (29.45)	84 (32.56)	147 (34.43)	
Number of sexual partners in the past 12 months (*n* = 847)					
0 sexual partner	56 (6.58)	18 (11.04)	19 (7.36)	19 (4.46)	0.042
1 sexual partner	616 (72.39)	104 (63.80)	185 (71.71)	325 (76.29)	
2 sexual partners	87 (10.22)	22 (13.50)	26 (10.08)	39 (9.15)	
3 or more	92 (10.81)	19 (11.66)	28 (10.85)	43 (10.09)	
Currently using contraception (*n* = 993)					
Yes	493 (49.45)	80 (33.89)	145 (46.77)	267 (59.73)	<0.001
No	504 (50.55)	156 (66.10)	165 (55.74)	180 (39.05)	
Ever pregnant (*n* = 800)					
Yes	370 (44.15)	27 (16.77)	111 (44.40)	228 (53.90)	<0.001
No	468 (55.85)	134 (83.23)	139 (55.60)	195 (46.10)	

### Data analysis

Exploratory factor analysis (EFA) was used to explore the factor structure and inform scale creation for the practical, procedural, and provider barrier domains. Based on *a priori* determination, we set a minimum eigenvalue of 1 and a factor loading cutoff of 0.4.^16^ EFA indicated single-factor solutions with full retention for both practical and procedural barriers. Thus, the practical barriers score is the mean of six items (Supplementary Table S1), with items 1, 3, 5, and 6 reverse coded. Similarly, the procedural barriers scale is the mean of seven items (Supplementary Table S2), with item 2 reverse coded. Cronbach’s alpha for the practical and procedural barriers in this sample were 0.80 and 0.91 respectively.

The EFA for provider barriers also indicated a one-factor solution. However, item 3 “My provider is judgmental of my outward appearance or weight” and item 7 “My provider pressures me toward a certain contraceptive method” had factor loadings <0.40 and were dropped from the scale. The scale was created by averaging the remaining 19 items. Items 1–9, 12, 14, and 18–21 were reverse scored. Cronbach’s alpha for the provider barriers scale in this sample was 0.94.

For all three barrier scales, a higher numerical score corresponded to a higher expressed barrier. We examined the utility of a scale for financial barriers; however, the factor loading for no insurance was <0.40. Because a scale requires more than two items to be useful, the three financial barrier items were examined as separate variables.^17^

Data were analyzed using SAS 9.4 (SAS Institute, Cary, NC, USA). Descriptive statistics, including means and proportions were calculated. Differences in means were compared using analysis of variance. Differences in proportions were compared using the chi-squared test. Logistic regression was used to identify the impact of practical barriers after adjusting for gender, race/ethnicity, sexual orientation, marital status, income, and financial barriers. Finally, logistic regression was used to model the relative impact of the various barrier scales (practical, procedural, provider) and financial barrier items on the timing of WWE, among those who had ever had a WWE.

## Results

Table 1 lists sample characteristics by WWE status. Briefly, the mean age was 24.5 years (standard deviation [SD] = 3.61). Most were cisgender female (90.8%) and White, non-Hispanic (63.4%). Approximately one-third (31.8%) were current students, and nearly half (48.6%) had never been married. Most reported having a primary care provider (81.0%), routine medical examination in the last year (69.6%), and health insurance (89.3%).

Approximately one-quarter (23.8%) reported never having a WWE, while 46.4% reported having an exam within the last year (on-time) and ∼30% (29.9%) reported their most recent exam >1 year ago (delayed). Sample characteristics differed by WWE status for all sociodemographic variables except gender. Specifically, those who never had a WWE were on average younger (average age 22.2), less likely to be married, more likely to be a minority race or ethnicity, more likely to be students, less likely to have a primary care provider, and less likely to have insurance.

Table 2 provides sexual and reproductive health characteristics by WWE status. Most respondents were heterosexual (72.2%) and reported having had a sexual experience with another person (85.2%). Among the sexually experienced, most (72.4%) reported one sexual partner in the previous year. Approximately half (49.5%) reported currently using contraception. Those who had never had a WWE were more likely to report a minority sexual orientation, less likely to be sexually experienced, less likely to have had at least one sexual partner in the past year, and less likely to have experienced a pregnancy. Those with on-time exams were most likely to have not had a period in the last 6 months and more likely to be using contraception. Among those who had ever had a WWE, 29.4% were 18–19 years of age at first exam, 21.2% were 16–17 years, and 20.7% were 20–21 years at first exam. Fewer than 15% of respondents who had ever had a WWE were less than 15 years or older than 22 years at the time of their first exam.

**Table 3.** Provider Experience Barriers by Screening Status Among Young Adult U.S. Females Who Have Ever Had a Well-Woman Exam

	Women who had ever had a well-woman exam					Delayed	On-time	
	Strongly agree (5)	Agree (4)	Neither agree nor disagree (3)	Disagree (2)	Strongly disagree (1)			
Variable	*n* (%)					Mean (SD)		*p*
Positively valenced questions—higher mean scores indicate LOWER barriers^a^								
1. My provider does not judge me because of my sexual activity (*n* = 693)	364 (52.53)	252 (36.36)	55 (7.94)	16 (2.31)	6 (0.87)	4.21 (0.89)	4.47 (0.72)	<0.001
2. My provider does not judge me for my lifestyle (691)	346 (50.07)	256 (37.05)	57 (8.25)	26 (3.76)	6 (0.87)	4.13 (0.93)	4.43 (0.77)	<0.001
4. My provider genuinely listens to my concerns (*n* = 691)	310 (44.86)	260 (37.63)	74 (10.71)	34 (4.92)	13 (1.88)	3.93 (1.01)	4.34 (0.87)	<0.001
5. My provider explains birth control options in a fair and unbiased way (*n* = 691)	321 (46.45)	255 (36.90)	64 (9.26)	33 (4.78)	18 (2.60)	4.00 (1.04)	4.31 (0.91)	<0.001
6. My provider explains tests and procedures well (*n* = 757)	358 (47.29)	314 (41.48)	64 (8.45)	17 (2.25)	4 (0.53)	4.15 (0.82)	4.44 (0.71)	<0.001
8. My provider respects my preference for birth control (*n* = 756)	334 (44.18)	277 (36.64)	96 (12.70)	32 (4.23)	17 (2.25)	3.95 (1.00)	4.30 (0.91)	<0.001
9. My provider explains the purpose of cancer screening well (*n* = 757)	300 (39.63)	288 (38.04)	124 (16.38)	35 (4.62)	10 (1.32)	3.83 (0.98)	4.27 (0.84)	<0.001
12. My provider spends adequate time with me to address my concerns (*n* = 757)	297 (39.23)	293 (38.71)	96 (12.68)	49 (6.47)	22 (2.91)	3.85 (1.05)	4.18 (0.98)	<0.001
14. My provider treats me with respect (*n* = 756)	394 (52.12)	284 (37.57)	59 (7.80)	13 (1.72)	6 (0.79)	4.24 (0.82)	4.48 (0.72)	<0.001
15. I can be open with my provider about sensitive topics (*n* = 757)	345 (45.57)	285 (37.65)	80 (10.57)	30 (3.96)	17 (2.25)	3.95 (1.04)	4.37 (0.83)	<0.001
18. I am provided adequate privacy at my appointment (*n* = 754)	385 (51.06)	294 (38.99)	47 (6.23)	17 (2.25)	11 (1.46)	4.21 (0.84)	4.46 (0.79)	<0.001
19. I feel that my provider attempted to make me as comfortable as possible (*n* = 757)	389 (51.39)	276 (36.46)	62 (8.19)	21 (2.77)	9 (1.19)	4.16 (0.87)	4.46 (0.79)	<0.001
20. My provider gives me adequate warning before touching sensitive areas of my body (*n* = 757)	371 (49.01)	293 (38.71)	59 (7.79)	26 (3.43)	8 (1.06)	4.17 (0.85)	4.41 (0.82)	<0.001
21. I feel at any point during examination, I can communicate to the provider that I do not wish to continue with the exam (*n* = 753)	338 (44.89)	243 (32.27)	105 (13.94)	47 (6.24)	20 (2.66)	3.87 (1.07)	4.26 (0.98)	**<**0.001
Negatively valenced questions—higher mean scores indicate HIGHER barriers								
3. My provider is judgmental of my outward appearance or weight (*n* = 689) (dropped from scale)	91 (13.21)	76 (11.03)	90 (13.06)	175 (25.40)	257 (37.30)	2.49 (1.38)	2.31 (1.43)	0.111
7. My provider pressures me toward a certain contraceptive method (*n* = 757) (dropped from scale)	78 (10.30)	108 (14.27)	82 (10.83)	217 (28.67)	272 (35.93)	2.33 (1.25)	2.35 (1.43)	0.884
10. My provider does not explain the risks or effects of common STIs (757)	40 (5.28)	71 (9.38)	127 (16.78)	257 (33.95)	262 (34.61)	2.33 (1.15)	2.06 (1.15)	<0.01
13. My provider seems to rush through our visit (*n* = 756)	396 (5.16)	69 (9.13)	116 (15.34)	277 (36.64)	255 (33.73)	2.30 (1.17)	2.06 (1.11)	<0.01
11. My provider is often distracted during my appointment (*n* = 755)	22 (2.91)	34 (4.50)	76 (10.07)	270 (35.76)	353 (46.75)	1.94 (0.98)	1.73 (0.98)	<0.01
17. My provider treats me differently because of my sexual orientation (*n* = 757)	20 (2.64)	24 (3.17)	61 (8.06)	210 (27.74)	442 (58.39)	1.70 (0.91)	1.60 (0.97)	0.164
16. My provider treats me differently because of my race (*n* = 758)	16 (2.11)	30 (3.96)	52 (6.86)	211 (27.84)	449 (59.23)	1.64 (0.85)	1.61 (0.98)	0.698

^a^ *p*-Value from *t*-test for the difference in means.

^b^ Individual items are in their original scale 1 = strongly disagree to 5 = strongly agree in this table. Positively valenced items are reverse coded when averaged into a scale.

STI, sexually transmitted infection.

Among all respondents, 37.2% had not received medical care in the past year due to financial concerns and 29% reported difficulty paying for medical expenses (Table 1). Those who had never had a WWE or with a delayed WWE were more likely to report that they had not received needed health care due to cost concerns relative to those with on-time WWE (Table 1). Similarly, those with delayed WWE were most likely to report difficulty paying medical expenses in the last year relative to the never and on-time groups (Table 1).

Among insured respondents, 25.7% expected no co-pay, 15.3% expected a co-pay of $1-$19, 20.7% expected a co-pay of $20-$29, 25.4% $30 or more, and 12.8% of respondents did not know what their expected co-pay would be. Among those who expected a co-pay, the majority (57.6%) reported that their co-pay would be “not at all” difficult to pay. Among those without insurance, 13.4% expected out-of-pocket costs to be $0-$49, 22%, 25.6%, 13.4%, and 25.6% expected their out-of-pocket costs to be $50-$99, $100-$149, $150-$199, and >$200 respectively. The majority (91.3%) reported that it would be at least somewhat difficult to meet their out-of-pocket costs.

Supplementary Table S1 displays overall agreement with practical barriers items and mean item agreement by WWE status. Those with on-time WWE expressed greater agreement that they could navigate these logistics, while those who had never had a WWE or with delayed WWE expressed greater agreement that they would encounter difficulties in finding a provider or arranging their schedule to attend an appointment. The mean practical barriers scale score for all respondents was 2.01 (SD = 0.71). After adjusting for sociodemographic factors, a single point increase in the practical barriers scale was associated with double the odds of never having a WWE (vs. on-time WWE) (OR: 2.26; 95% CI: 1.86–2.74).

Those who had ever had a WWE (*n* = 763) were asked which part of the exam contributed most to fear or discomfort during the visit. Almost half (47.2%) reported pap smear testing contributed most to fear or discomfort, followed by the pelvic exam (19.7%). Only 5% reported that the breast exam contributed most to fear or discomfort and 26.7% reported that they did not experience fear or discomfort during exams. Those with on-time WWE (31.4%) were more likely to report not experiencing fear/discomfort than those with delayed WWE (19.9%; *p* < 0.01).

Supplementary Table S2 shows the percentage who indicated each level of agreement with statements related to each procedural barrier, among those who had ever had a WWE. Additionally, a comparison of the mean procedural barrier scores by exam status is provided. Among those who had ever had a WWE, the highest proportion of respondents agreed that they experience “high levels of stress” during speculum insertion, followed closely by lying on the exam table with feet in stirrups, and manual pelvic examination. The fewest respondents agreed they experienced “high levels of stress” having to undress at the gynecologist. Most agreed that they were given adequate privacy during appointments (90.1%) and that their provider attempted to make them as comfortable as possible (87.9%). Mean scores for all negatively valenced items were higher for those with delayed WWE, whereas mean scores for all positively valenced items were higher for those with on-time WWE. The mean procedural barrier scale score for those who had ever had a WWE was 2.48 (SD = 0.78).

Provider experience barriers among those who ever had a WWE are presented in Table 3. Across all items, most reported positive experiences with providers including that they did not feel judged, felt their providers listened, and explained exams and results well. However, those with delayed WWE had lower mean scores on items regarding providers explaining tests and procedures well, respecting contraceptive preferences, explaining the purpose of cancer screening, being treated with respect, and ability to be open with providers. Similarly, while most disagreed or strongly disagreed with negatively valenced items, those with delayed WWE had higher mean scores for items regarding providers feeling rushed or being distracted. The mean provider barrier scale score for those who had ever had a WWE was 1.91 (SD = 0.68).

Among those with delayed WWE the most highly endorsed reasons for not having an exam in the last 12 months were discomfort being naked in front of providers (70.7% agreed or strongly agreed), being uncomfortable talking to providers about sexual health (44.0% agreed or strongly agreed) and being self-conscious about odors or cleanliness (57.7% agreed or strongly agreed). Full results for these items are presented in Supplementary Table S3.

A total of 205 respondents reported that they intentionally delayed recommended pap smear testing. The most endorsed reasons for delaying pap testing were fear the exam would be painful, discomfort being naked in front of providers, and discomfort with provider touching sensitive areas (Supplementary Table S4). A total of 79 respondents reported they intentionally delayed recommended STI testing. The most endorsed reasons for delaying STI testing in this group were discomfort being naked in front of providers, difficulty discussing sensitive topics, or fear of finding something incurable (Supplementary Table S5).

We examined whether barriers varied by race/ethnicity, gender identity, and sexual orientation. Supplementary Table S6 provides the full results of these analyses. Persons with a nonfemale gender, a sexual orientation of “something else” (vs. heterosexual) and those who were “other single race” (vs. White, non-Hispanic) reported higher practical barriers. Mean provider barriers differed for all three sociodemographic variables with those who reported a nonfemale gender, a sexual orientation of gay/lesbian or “something else” or Hispanic ethnicity (vs. White, non-Hispanic) reporting higher provider barriers. The three financial barriers and the procedural barriers did not differ by these sociodemographic factors (data not shown).

In unadjusted models, practical, procedural, and provider barriers as well as lack of insurance and delayed medical care in the last year due to cost concerns were significantly associated with delayed WWE (Table 4). When adjusting for other barrier types, only lack of insurance, procedural barriers, and provider-related barriers were significantly associated with delayed WWE. These associations remained significant after adjustment for sociodemographic variables. In adjusted models, lack of insurance was associated with three times the odds of delayed WWE (OR: 3.07 95% CI: 1.59–5.98), a one-point increase in the procedural barriers was associated with a 22% increase in the odds of delayed WWE (OR: 1.22; 95% CI: 1.03–1.46) and a one-point increase in the provider-related barriers scale was associated with >50% increased odds of delayed WWE (OR: 1.54; 95% CI: 1.14–2.08).

**Table 4.** Logistic Regression Analysis for the Association Between Practical, Procedural, Provider, and Financial Barriers and Delayed Well-Woman Exam in a Sample of Young Adult Females in the United States

		Delayed well-woman exam	
	Unadjusted models	Barriers base model	Adjusted model^a^
	OR (95% CI)		
Practical barriers	1.68 (1.33–2.12)	1.25 (0.93–1.68)	1.18 (0.87–1.61)
Procedural barriers	1.41 (1.21–1.64)	1.25 (1.06–1.48)	1.22 (1.03–1.46)
Provider barriers	2.02 (1.60–2.56)	1.51 (1.14–2.02)	1.54 (1.14–2.08)
Financial barriers			
No Insurance	2.97 (1.68–5.26)	2.99 (1.58–5.68)	3.07 (1.59–5.98)
Difficulty paying for medical expenses in the last 12 months	1.25 (0.92–1.72)	1.06 (0.71–1.57)	1.01 (0.67–1.52)
Delayed needed care due to cost concerns in the last 12 months	1.54 (1.13–2.08)	1.09 (0.73–1.63)	1.09 (0.71–1.65)

^a^ Adjusted for age, gender identity, race/ethnicity, sexual orientation, marital status, and income.

## Discussion

Those who never had a WWE were younger, less likely to be married, had less education, lower income, and were more likely to be Hispanic and multiracial than the overall sample. This is consistent with pelvic examination rates in the prior 12 months as reported by the NSFG, which found the lowest screening rate among Hispanic females and that pelvic exam rates are positively correlated with increasing education and income.^18^ Additionally, those who had never had a WWE were more likely to report minority sexual orientation. Reduced rates of WWE among those with minority sexual orientations may be influenced by less demand for contraception in same-sex relationships, a perceived lack of need for WWE, or increased barriers within the health care environment for nonheterosexuals.^19^

Despite nearly 90% of the sample reporting they had insurance, almost 40% reported that they had delayed medical care due to cost concerns and 29% reported they had difficulty paying for medical expenses in the past year. These percentages are higher than those observed in a 2022 Kaiser Family Foundation Analysis of NHIS data which found that 28% of adults of all ages had delayed or not received medical care in the prior year, due to cost and 11% had difficulty paying medical bills.^20^ The higher percentage in our sample is likely attributable to differences in age as we focused only on persons 18–30 years old who are more likely to be students, and less likely to have stable income sources.

Over the last two decades, rates of health insurance have increased substantially such that >90% of U.S. adults were covered by health insurance in 2022.^21^ However, lack of health insurance and the cost of health care, even with insurance coverage, remain significant barriers to seeking health care.^22^ In our adjusted logistic regression models, lack of health insurance was associated with the highest odds of delayed WWE. Since the passage of the Affordable Care Act (ACA), preventive health care has become increasingly accessible. Under the ACA, WWEs must be provided to insured patients without co-pays or cost-sharing. Indeed, a 2017 study found that the percentage of females who reported delaying or skipping needed care because of costs fell to an all-time low.^23^ Interestingly, in our sample, nearly three-fourths of insured patients expected a co-pay for a well-woman visit. While most who expected a co-pay did not report perceived difficulties in meeting the co-pay, 42.4% expected that they would have some difficulty in meeting the co-pay. This may suggest that provisions of the ACA related to preventive coverage without cost-sharing are not well understood among young adult females in the United States. Research from the early post-ACA period found low consumer awareness of the no-cost-sharing provisions of the ACA.^24^ Our finding may indicate that lower awareness persists, particularly in relatively young adults, who may be newer to independently navigating the health care system. A 2021 study of low-income females identified that 72% perceived financial costs associated with cervical cancer screening.^25^ Additional research is needed to understand why this false perception exists and whether perceived copays for preventive care are associated with delayed or missed preventive health care.

Those who had never had a WWE were most likely to report practical barriers to health care such as transportation and difficulty finding a provider. Even after accounting for insurance status and other sociodemographic factors, practical barriers with associated with twice the odds of never having a WWE. Difficulties navigating the health care system and with transportation are well-established barriers to health care that disproportionately affect low-income and marginalized communities.^26,27^

Although most respondents reported positive experiences with providers, increased provider-related barriers were associated with 54% increased odds of delayed WWE. This suggests that a patient’s experience with their provider is an important factor in continued and on-time preventive care. This is consistent with other findings indicating that provider communication (one aspect of provider experience) is important in the uptake of preventive care.^28^ Our results showed a high correlation between physical discomfort during examination and delayed care. These findings were supported by O’Laughlin and colleagues, who reported that anxiety and fear surrounding pelvic examination hinder compliance with preventive health screenings, and result in delayed or avoided care with significant health consequences such as delayed detection or treatment for infections or cancers.^13^

Although most reported on-time WWE, we found that White, non-Hispanic participants were the most likely to report they had intentionally delayed pap smear screening. This aligns with some previous studies on cervical cancer screening which found that Black, non-Hispanic and Hispanic females are less likely to delay cervical cancer screening compared to White, non-Hispanics.^6,29^ It is important to note that our outcome of interest was an annual WWE. With advances in cervical cancer screening, annual screening is no longer recommended for low-risk females. Current American College of Obstetrics and Gynecology guidelines recommend cervical cytology screening every 3 years for those in the age group of our sample. However, our survey did not define whether someone was overdue for a pap smear test, but rather respondents were asked to self-report intentional delay of pap smear testing and STI screening.

Notably, the rate of reported contraceptive use in our sample was lower than recent national estimates dating from 2017 to 2019 data.^30^ Research has demonstrated that contraceptive use declined during the COVID-19 pandemic.^31^ Our data were collected in 2022 after most health care had returned to prepandemic availability.^32^ However, it is possible that pandemic-related decreases in contraception persisted for some in the sample. Additionally, approximately 15% of the sample reported they were not sexually experienced and 3% reported they were currently pregnant. We did not evaluate fertility intentions, but it is likely that some subset of our sample was trying to conceive. Further, it is possible that this represents changes in contraceptive use in this population. Young adults report that social media plays an increasingly important source of sexual health information and research indicates that social media content is largely negative about hormonal contraceptive methods.^33,34^ Additional research is needed to understand if contraceptive use patterns are changing in young adult females.

### Strengths and limitations

This study examined care barriers across four domains (financial, practical, procedural, and provider) in a large sample of young adult females. However, the study was cross-sectional and relied on recalled information about provider experience. Although we used quota sampling to improve the representativeness of the sample, our sample may not be a fully representative and may not accurately represent the experience and barriers of all young adult females in the United States. We had an insufficient sample size of gender minorities to adequately examine the barriers to WWE among gender minorities.

## Conclusions

A 2018 report on The Well-Woman Project, by Handler et al. found among its main themes that relationships with providers are key to female’s decisions to access health care.^24^ Our findings support the value and importance of the patient−provider relationship in supporting on-time preventive health care. Unlike significant financial and practical barriers to care, the patient−provider experience can be influenced by providers. It is important to note that most respondents had positive experiences. Providers should be encouraged to continue to construct positive care environments. Specific attention to the acceptability of care environments for marginalized populations is warranted. The nature of gynecological care is inherently sensitive. Research indicates that providers can reduce anxiety during pelvic exams by educating patients about the procedure, offering a chaperone or support person, ensuring comfort with proper speculum size, lubrication, draping, and positioning, and avoiding dismissive or invalidating communication. Providers may also improve patient satisfaction by validating patient’s physical or emotional discomfort through empathetic listening training.^35,36^

## Abbreviations Used

ACA: Affordable Care Act
BRFSS: Behavioral Risk Factor Surveillance System
CI: Confidence Interval
EFA: Exploratory Factor Analysis
NHIS: National Health Interview Survey
NSFG: National Survey of Family Growth
OR: Odds Ratio
SD: Standard Deviation
STI: Sexually Transmitted Infection
WWE: Well Woman Exam

## References

[1] ACOG Committee opinion no. 754: The utility of and indications for routine pelvic examination. Obstet Gynecol 2018;132(4):e174–e180; doi: 10.1097/AOG.0000000000002895 30247363

[2] Get Your Well-Woman Visit Every Year. 2024. Available from: https://health.gov/myhealthfinder/healthy-living/sexual-health/get-your-well-woman-visit-every-year [Last accessed: August 2, 2024].

[3] ACOG Committee Opinion no. 755: Well-woman visit. Obstet Gynecol 2018;132(4):e181–e186; doi: 10.1097/AOG.0000000000002897 30247364

[4] Mathews TJ, Hamilton BE. Mean age of mothers is on the rise: United States, 2000-2014. NCHS Data Brief 2016;232(232):1–8. 26828319

[5] Koh B, Tan DJH, Ng CH, et al. Patterns in cancer incidence among people younger than 50 years in the US, 2010 to 2019. JAMA Netw Open 2023;6(8):e2328171; doi: 10.1001/jamanetworkopen.2023.28171 37585204 PMC10433086

[6] Francoeur AA, Liao CI, Caesar MA, et al. The increasing incidence of stage IV cervical cancer in the USA: What factors are related? Int J Gynecol Cancer 2022;32(9):1115–1122; doi: 10.1136/ijgc-2022-003728 35981903

[7] Cancer Facts & Figures 2024. Atlanta, GA, USA; 2024. Available from: https://www.cancer.org/cancer/types/cervical-cancer/detection-diagnosis-staging/survival.html [Last accessed: August 2, 2024].

[8] Lawrence EM, Mollborn S, Hummer RA. Health lifestyles across the transition to adulthood: Implications for health. Soc Sci Med 2017;193:23–32; doi: 10.1016/j.socscimed.2017.09.041 28992538 PMC5659920

[9] Racial and Ethnic Diversity in the United States. 2010 Census and 2020 Census. 2021. Available from: https://www.census.gov/library/visualizations/interactive/racial-and-ethnic-diversity-in-the-united-states-2010-and-2020-census.html [Last accessed: August 2, 2024].

[10] 2017-2019 National Survey of Family Growth Public-Use Data and Documentation. CDC National Center for Health Statistics: Hyattsville, MD; 2020.

[11] Behavioral Risk Factor Surveillance System 2020 Questionnaire. 2020. Available from: https://www.cdc.gov/brfss/questionnaires/pdf-ques/2020-BRFSS-Questionnaire-508.pdf [Last accessed: August 2, 2024].

[12] National Health Interview Public-Use File Codebook. 2021. Available from: https://ftp.cdc.gov/pub/Health_Statistics/NCHS/Dataset_Documentation/NHIS/2021/adult-codebook.pdf [Last accessed: August 2, 2024].

[13] O’Laughlin DJ, Strelow B, Fellows N, et al. Addressing anxiety and fear during the female pelvic examination. J Prim Care Community Health 2021;12:2150132721992195; doi: 10.1177/2150132721992195 33525968 PMC7970676

[14] Janssen SM, Lagro-Janssen AL. Physician’s gender, communication style, patient preferences and patient satisfaction in gynecology and obstetrics: A systematic review. Patient Educ Couns 2012;89(2):221–226; doi: 10.1016/j.pec.2012.06.034 22819711

[15] Okoro ON, Hillman LA, Cernasev A. “We get double slammed!”: Healthcare experiences of perceived discrimination among low-income African-American women. Womens Health (Lond) 2020;16:1745506520953348; doi: 10.1177/1745506520953348 32856564 PMC7457641

[16] Tavakol M, Wetzel A. Factor analysis: A means for theory and instrument development in support of construct validity. Int J Med Educ 2020;11:245–247; doi: 10.5116/ijme.5f96.0f4a 33170146 PMC7883798

[17] Raubenheimer J. An item selection procedure to maximise scale reliability and validity. Journal of Industrial Psychology 2004;30(4):59–64.

[18] Martinez GM, Qin J, Saraiya M, et al. Receipt of pelvic examinations among women aged 15-44 in the United States, 1988-2017. NCHS Data Brief 2019;339(339):1–8. 31442190

[19] Hsieh N, Shuster SM. Health and health care of sexual and gender minorities. J Health Soc Behav 2021;62(3):318–333; doi: 10.1177/00221465211016436 34528481

[20] Rakshit S, Amin K, Cox C. How does cost affect access to healthcare? 2024. Available from: https://www.healthsystemtracker.org/chart-collection/cost-affect-access-care/#Percent%20of%20adults%20who%20reported%20barriers%20to%20accessing%20medical%20care,%202022 [Last accessed: August 23, 2024].

[21] Keisler-Starkey K, Bunch LN, Lindstrom RA. Health Insurance Coverage in the United States: 2022. U.S. Government Publishing Office: Washington, D.C.; 2023.

[22] Hawks L, Himmelstein DU, Woolhandler S, et al. Trends in unmet need for physician and preventive services in the United States, 1998-2017. JAMA Intern Med 2020;180(3):439–448; doi: 10.1001/jamainternmed.2019.6538 31985751 PMC6990729

[23] Gunja MZ, Collins SR, Doty MM, et al. How the affordable care act has helped women gain insurance and improved their ability to get health care: Findings from the commonwealth fund biennial health insurance survey, 2016. Issue Brief (Commonw Fund) 2017;1–18. 28805362

[24] Handler A, Henderson V, Johnson R, et al. The well-woman project: Listening to women’s voices. Health Equity 2018;2(1):395–403; doi: 10.1089/heq.2018.0031 30623168 PMC6323588

[25] Biddell CB, Spees LP, Smith JS, et al. Perceived financial barriers to cervical cancer screening and associated cost burden among low-income, under-screened women. J Womens Health (Larchmt) 2021;30(9):1243–1252; doi: 10.1089/jwh.2020.8807 33851854 PMC8558088

[26] Griese L, Berens EM, Nowak P, et al. Challenges in navigating the health care system: Development of an instrument measuring navigation health literacy. Int J Environ Res Public Health 2020;17(16); doi: 10.3390/ijerph17165731 PMC7460304 32784395

[27] Henning-Smith C. The public health case for addressing transportation-related barriers to care. Am J Public Health 2020;110(6):763–764; doi: 10.2105/AJPH.2020.305638 32374700 PMC7204464

[28] Oh NL, Biddell CB, Rhodes BE, et al. Provider communication and HPV vaccine uptake: A meta-analysis and systematic review. Prev Med 2021;148:106554; doi: 10.1016/j.ypmed.2021.106554 33857561

[29] Zeno EE, Brewer NT, Spees LP, et al. Racial and ethnic differences in cervical cancer screening barriers and intentions: The My Body My Test-3 HPV self-collection trial among under-screened, low-income women. PLoS One 2022;17(10):e0274974; doi: 10.1371/journal.pone.0274974 36227948 PMC9562154

[30] Daniels K, Abma JC. Current contraceptive status among women aged 15-49: United States, 2017–2019. NCHS Data Brief 2020(388):1–8. 33151146

[31] Steenland MW, Geiger CK, Chen L, et al. Declines in contraceptive visits in the United States during the COVID-19 pandemic. Contraception 2021;104(6):593–599; doi: 10.1016/j.contraception.2021.08.003 34400152 PMC8570647

[32] Mehrotra A, Chernew ME, Linetsky D, et al. The impact of COVID-19 on outpatient visits in 2020: Visits remained stable, despite a late surger in cases. 2021. Available from: https://www.commonwealthfund.org/publications/2021/feb/impact-covid-19-outpatient-visits-2020-visits-stable-despite-late-surge [Last accessed: March 19, 2025].

[33] Pfender EJ, Devlin MM. What do social media influencers say about birth control? A content analysis of YouTube vlogs about birth control. Health Commun 2023;38(14):3336–3345; doi: 10.1080/10410236.2022.2149091 36642835

[34] Schneider-Kamp A, Takhar J. Interrogating the pill: Rising distrust and the reshaping of health risk perceptions in the social media age. Soc Sci Med 2023;331:116081; doi: 10.1016/j.socscimed.2023.116081 37441974

[35] Hildenbrand GM, Perrault EK, Rnoh RH. Patients’ perceptions of health care providers’ dismissive communication. Health Promot Pract 2022;23(5):777–784; doi: 10.1177/15248399211027540 34344222

[36] Bates CK, Carroll N, Potter J. The challenging pelvic examination. J Gen Intern Med 2011;26(6):651–657; doi: 10.1007/s11606-010-1610-8 21225474 PMC3101979

