# Supplementary material for: Barriers to Routine Gynecological Care in Young Adult Females in the United States
Source: Womens Health Rep (New Rochelle). 2025 May 19;6(1):586–98. doi: 10.1089/whr.2025.0015 (PMC12177321; doi:10.1089/whr.2025.0015)
Supplement: Supplementary Table S1 [file whr.2025.0015_supplementary_table_s1.docx]

**Supplemental Table 1. Respondent agreement with statements related to the practical barriers of seeing a healthcare provider by screening status in a sample of young adult females in the U.S.**

|  | Total Sample | | | | | Never | Delayed | Ontime |  |
| --- | --- | --- | --- | --- | --- | --- | --- | --- | --- |
|  | Strongly Agree  **(5)** | Agree  **(4)** | Neither Agree nor Disagree  **(3)** | Disagree  **(2)** | Strongly Disagree  **(1)** | n = 237 | n = 297 | n = 462 |  |
| Variable | n (%) | | | | | Mean (SD) | | | p-value^a^ |
| Positively valenced questions – higher mean scores indicate LOWER barriers.^b^ | | | | | | | | | |
| 1. Confident I could find a gynecological care provider (n=1000) | 481 (48.10) | 377 (37.70) | 87 (8.70) | 42 (4.20) | 13 (1.30) | 3.79 (1.07) | 4.23 (0.83) | 4.54 (0.69) | **<0.001** |
| 3. I am confident I know how to make an appointment with a gynecological care provider (n=999) | 532 (53.25) | 293 (29.33) | 85 (8.51) | 64 (6.41) | 25 (2.50) | 3.50 (1.24) | 4.32 (0.88) | 4.57 (0.75) | **<0.001** |
| 5. I am confident I could arrange my schedule to attend an appointment with a gynecological care provider (n=1000) | 353 (35.30) | 378 (37.80) | 140 (14.00) | 101 (10.10) | 28 (2.80) | 3.64 (1.10) | 3.91 (1.04) | 4.08 (1.05) | **<0.001** |
| 6. Confident I could find transportation to reach the clinic (n=998) | 545 (54.61) | 324 (32.46) | 72 (7.21) | 42 (4.21) | 15 (1.50) | 4.08 (1.02) | 4.36 (0.83) | 4.47 (0.85) | **<0.001** |
| Negatively valenced questions – higher mean scores indicate HIGHER barriers | | | | | | | | | |
| 2. It would be difficult to arrange my schedule to attend an appointment with a gynecological care provider (n=1000) | 87 (8.70) | 203 (20.30) | 209 (20.90) | 366 (36.90) | 135 (13.50) | 2.90 (1.11) | 2.78 (1.20) | 2.64 (1.19) | **<0.05** |
| 4. It would be difficult to find a gynecological care provider (n=999) | 22 (2.20) | 78 (7.81) | 180 (18.02) | 416 (41.64) | 303 (30.33) | 2.54 (1.04) | 2.13 (0.96) | 1.86 (0.91) | **<0.001** |

**a: p-value from ANOVA test for the difference in means
b: Individual items are in their original scale 1 = strongly disagree to 5 = strongly agree in this table. Positively-valenced items are reverse coded when averaged into a scale.**
