# Supplementary material for: Barriers to Routine Gynecological Care in Young Adult Females in the United States
Source: Womens Health Rep (New Rochelle). 2025 May 19;6(1):586–98. doi: 10.1089/whr.2025.0015 (PMC12177321; doi:10.1089/whr.2025.0015)
Supplement: Supplementary Table S2 [file whr.2025.0015_supplementary_table_s2.docx]

**Supplemental Table 2: Procedural experience barriers by screening status among young adult U.S. females who have ever had a well-woman exam.**

|  | Women who had ever had a well-woman exam | | | | | Delayed | On-time |  |
| --- | --- | --- | --- | --- | --- | --- | --- | --- |
|  | **Strongly Agree**  **(5)** | **Agree**  **(4)** | **Neither Agree nor Disagree**  **(3)** | **Disagree**  **(2)** | **Strongly Disagree**  **(1)** |  |  |  |
| **Variable** | n (%) | | | | | Mean (SD) | | p-value^a^ |
| Negatively valenced questions – higher mean scores indicate HIGHER barriers | | | | | | | | |
| 5. I experience high levels of stress during the insertion of the speculum into the vagina (n=705) | 167 (23.69) | 224 (31.77) | 92 (13.05) | 135 (19.15) | 87 (12.34) | 3.58 (1.24) | 3.23 (1.40) | **<0.001** |
| 3. I experience high levels of stress while lying on the examination table with feet in stirrups (n=705) | 150 (21.28) | 182 (25.82) | 121 (17.16) | 154 (21.84) | 98 (13.90) | 3.39 (1.27) | 3.07 (1.40) | **<0.01** |
| 4. I experience high levels of stress during the manual pelvic examination (n=705) | 144 (20.43) | 174 (24.68) | 128 (18.16) | 161 (22.84) | 98 (13.90) | 3.41 (1.27) | 3.00 (1.38) | **<0.001** |
| 1. I experience high levels of stress when having a Pap Smear examination (n=707) | 111 (15.70) | 150 (21.22) | 151 (21.36) | 168 (23.76) | 127 (17.96) | 3.20 (1.26) | 2.78 (1.36) | **<0.001** |
| 6. When my provider inserted the speculum, it was very painful (n=704) | 74 (10.51) | 157 (22.30) | 154 (21.88) | 212 (30.11) | 107 (15.20) | 3.01 (1.15) | 2.73 (1.27) | **<0.01** |
| 7. I experience high levels of stress when having to undress at the gynecologist (n=706) | 72 (10.20) | 126 (17.85) | 126 (17.85) | 237 (33.57) | 145 (20.54) | 2.84 (1.23) | 2.51 (1.28) | **<0.001** |
| Positively valenced questions – higher mean scores indicate LOWER barriers^b^ | | | | | | | | |
| 2. I feel relatively at ease when my healthcare provider performs a physical examination (n=707) | 132 (18.67) | 233 (32.96) | 163 (23.06) | 128 (18.10) | 51 (7.21) | 3.08 (1.14) | 3.55 (1.18) | **<0.001** |

**a: p-value from t-test for the difference in means
b: Individual items are in their original scale 1 = strongly disagree to 5 = strongly agree in this table. Positively-valenced items are reverse coded when averaged into a scale.**
