# Supplementary material for: Barriers to Routine Gynecological Care in Young Adult Females in the United States
Source: Womens Health Rep (New Rochelle). 2025 May 19;6(1):586–98. doi: 10.1089/whr.2025.0015 (PMC12177321; doi:10.1089/whr.2025.0015)
Supplement: Supplementary Table S3 [file whr.2025.0015_supplementary_table_s3.docx]

**Supplemental Table 3: Reasons for delaying a well-woman exam among a sample of young adult U.S. females who had not had a well-woman exam in the past 12 months.**

| Variable | Mean (SD) | **Strongly Agree**  **(5)** | **Agree**  **(4)** | **Neither Agree nor Disagree**  **(3)** | **Disagree**  **(2)** | **Strongly Disagree (1)** |
| --- | --- | --- | --- | --- | --- | --- |
| I am nervous or uncomfortable about being naked in front of the providers (n=293) | 3.83 (1.28) | 114 (38.91) | 93 (31.74) | 32 (10.92) | 29 (9.90) | 25 (8.53) |
| I am uncomfortable talking to a provider about my sexual health (n=293) | 3.13 (1.33) | 57 (19.45) | 72 (24.57) | 53 (18.09) | 73 (24.91) | 38 (12.97) |
| I would be self-conscious of odor and cleanliness (n=293) | 3.37 (1.35) | 66 (22.53) | 103 (35.15) | 36 (12.29) | 48 (16.38) | 40 (13.65) |
| I have no health concerns (n=293) | 3.05 (1.18) | 30 (10.24) | 89 (30.38) | 71 (24.23) | 71 (24.23) | 32 (10.92) |
| I do not have a provider (n=293) | 3.08 (1.45) | 62 (21.16) | 73 (24.91) | 43 (14.68) | 56 (19.11) | 59 (20.14) |
| I am too busy (n=293) | 2.74 (1.21) | 25 (8.53) | 66 (22.53) | 55 (18.77) | 103 (35.15) | 44 (15.02) |
| I have had negative experiences with health care providers before (n=292) | 2.62 (1.25) | 24 (8.22) | 63 (21.58) | 40 (13.70) | 108 (36.99) | 46.57 (19.52) |
| I do not want to have my weight measured (n=293) | 2.61 (1.37) | 44 (15.02) | 37 (12.63) | 44 (15.02) | 97 (33.11) | 71 (24.23) |
| I am concerned I would not be able to pay for an exam (n=293) | 2.71 (1.41) | 45 (15.36) | 52 (17.75) | 45 (15.36) | 76 (25.94) | 75 (25.60) |
| I do not feel like a provider would listen to me (n=293) | 2.56 (1.11) | 19 (6.48) | 40 (13.65) | 73 (24.91) | 114 (38.91) | 47 (16.04) |
| I am not sexually active (n=293) | 2.82 (1.62) | 77 (26.28) | 39 (13.31) | 22 (7.51) | 63 (21.50) | 92 (31.40) |
| I am afraid of being sexually assaulted (n=291) | 2.67 (1.35) | 38 (13.06) | 48 (16.49) | 58 (19.93) | 75 (25.77) | 72 (24.74) |
| I have a history of sexual or other abuse (n=293) | 2.34 (1.37) | 26 (8.87) | 50 (20.33) | 19 (7.72) | 57 (23.17) | 102 (41.46) |
| I would not know how to find a provider (n=293) | 2.43 (1.19) | 19 (6.48) | 47 (16.04) | 44 (15.02) | 115 (39.25) | 68 (23.21) |
| I am wary of COVID-19 (n=293) | 2.18 (1.16) | 12 (4.10) | 36 (12.29) | 49 (16.72) | 93 (31.74) | 103 (35.15) |
| I do not have health insurance (n=293) | 2.09 (1.25) | 26 (8.87) | 18 (6.14) | 33 (11.26) | 95 (32.42) | 121 (41.30) |
| I do not want to have my blood pressure checked (n=293) | 1.86 (0.96) | 12 (4.10) | 6 (2.05) | 28 (9.56) | 131 (44.71) | 116 (39.59) |
| Religious beliefs (n=293) | 1.81 (1.01) | 10 (3.41) | 12 (4.10) | 31 (10.58) | 100 (34.13) | 140 (47.78) |
| I am living with a physical condition that makes it too hard to get to an appointment (n=293) | 1.72 (0.81) | 134 (35.73) | 122 (41.64) | 25 (8.53) | 10 (3.41) | 2 (0.68) |
| My immigration status makes me nervous to see a doctor (n=291) | 1.45 (0.7) | 1 (0.34) | 5 (1.72) | 15 (5.15) | 81 (27.84) | 189 (64.95) |
| I am unable to communicate with a provider due to a language barrier (n=293) | 1.43 (0.65) | 0 | 3 (1.02) | 16 (5.46) | 86 (29.35) | 188 (64.16) |
