# Supplementary material for: Barriers to Routine Gynecological Care in Young Adult Females in the United States
Source: Womens Health Rep (New Rochelle). 2025 May 19;6(1):586–98. doi: 10.1089/whr.2025.0015 (PMC12177321; doi:10.1089/whr.2025.0015)
Supplement: Supplementary Table S4 [file whr.2025.0015_supplementary_table_s4.docx]

**Supplemental Table 4: Reasons for intentionally delaying a pap smear among a sample of sexually experienced young adult U.S. females.**

| **Variable** | Mean (SD) | **Strongly Agree**  **(5)** | **Agree**  **(4)** | **Neither Agree nor Disagree**  **(3)** | **Disagree**  **(2)** | **Strongly Disagree**  **(1)** |
| --- | --- | --- | --- | --- | --- | --- |
| I am afraid it will hurt (n=204) | 4.14 (1.12) | 101 (49.51) | 64 (31.37) | 16 (7.84) | 13 (6.37) | 10 (4.90) |
| I am nervous or uncomfortable about being naked in front of the provider (n=204) | 3.84 (1.21) | 73 (35.78) | 76 (37.25) | 16 (7.84) | 27 (13.24) | 12 (5.88) |
| I am nervous or uncomfortable with the provider touching sensitive areas of my body (n=205) | 3.83 (1.17) | 65 (31.71) | 88 (42.93) | 16 (7.80) | 24 (11.71) | 12 (5.85) |
| I am afraid of the provider finding something wrong or incurable (n=204) | 3.78 (1.20) | 70 (34.31) | 68 (33.33) | 26 (12.75) | 31 (15.20) | 9 (4.41) |
| I am afraid of the provider being too rough/insensitive physically (n=205) | 3.54 (1.37) | 63 (30.73) | 64 (31.22) | 21 (10.24) | 34 (16.59) | 23 (11.22) |
| I have difficulty openly discussing sensitive topics with the provider (n=204) | 3.40 (1.33) | 49 (24.02) | 67 (32.84) | 27 (13.24) | 39 (19.12) | 22 (10.78) |
