# Supplementary material for: Barriers to Routine Gynecological Care in Young Adult Females in the United States
Source: Womens Health Rep (New Rochelle). 2025 May 19;6(1):586–98. doi: 10.1089/whr.2025.0015 (PMC12177321; doi:10.1089/whr.2025.0015)
Supplement: Supplementary Table S5 [file whr.2025.0015_supplementary_table_s5.docx]

**Supplemental Table 5: Reasons for intentionally delaying sexually transmitted infection testing among a sample of sexually experienced young adult U.S. females.**

| **Variable** | Mean (SD) | **Strongly Agree**  **(5)** | **Agree**  **(4)** | **Neither Agree nor Disagree**  **(3)** | **Disagree**  **(2)** | **Strongly Disagree**  **(1)** |
| --- | --- | --- | --- | --- | --- | --- |
| I am nervous or uncomfortable about being naked in front of the provider (n=79) | 3.58 (1.39) | 26 (32.91) | 25 (31.65) | 6 (7.59) | 13 (16.46) | 9 (11.39) |
| I have difficulty openly discussing sensitive topics with the provider (n=79) | 3.43 (1.37) | 20 (25.32) | 27 (34.18) | 10 (12.66) | 11 (13.92) | 11 (13.92) |
| I am afraid of the provider finding something wrong or incurable (n=79) | 3.23 (1.80) | 34 (43.04) | 8 (10.13) | 7 (8.86) | 2 (2.53) | 28 (35.44) |
| I am afraid it will hurt (n=78) | 3.13 (1.65) | 29 (37.18) | 3 (3.85) | 16 (20.51) | 9 (11.54) | 21 (26.92) |
| I am nervous or uncomfortable with the provider touching sensitive areas of my body (n=79) | 3.09 (1.59) | 24 (30.38) | 9 (11.39) | 18 (22.78) | 6 (7.59) | 22 (27.85) |
| I am afraid of the provider being too rough/insensitive physically (n=79) | 2.86 (1.47) | 18 (22.78) | 6 (7.59) | 22 (27.85) | 13 (16.46) | 20 (25.32) |
| I have a fear/aversion to taking medicine (n=78) | 2.31 (1.18) | 4 (5.13) | 11 (14.10) | 12 (15.38) | 29 (37.18) | 22 (28.21) |
