# Supplementary material for: Barriers to Routine Gynecological Care in Young Adult Females in the United States
Source: Womens Health Rep (New Rochelle). 2025 May 19;6(1):586–98. doi: 10.1089/whr.2025.0015 (PMC12177321; doi:10.1089/whr.2025.0015)
Supplement: Supplementary Table S6 [file whr.2025.0015_supplementary_table_s6.docx]

**Supplemental Table 6. Mean barrier scores by selected sociodemographic characteristics in a sample of young adult U.S. females.**

|  | Total Sample | | Females who had ever had a well-woman exam | | | |
| --- | --- | --- | --- | --- | --- | --- |
|  | Practical Barriers | | Procedural Barriers | | Provider Experience Barriers | |
|  | Mean (SD) | p-value | Mean (SD) | p-value | Mean (SD) | p-value |
| Gender Identity |  |  |  |  |  |  |
| **Cisgender female** | 1.99 (0.69) | **<0.01** | 2.47 (0.78) | **0.443** | 1.89 (0.66) | **0.033** |
| Something else | 2.20 (0.81) |  | 2.55 (0.82) |  | 2.05 (0.77) |  |
| Sexual Orientation (n=1000) |  |  |  |  |  |  |
| Heterosexual | 1.94 (0.66) | **<0.001** | 2.43 (0.76) | **<0.05^a^** | 1.87 (0.66) | **<0.001** |
| Bisexual | 2.10 (0.74) |  | 2.54 (0.79) |  | 1.93 (0.68) |  |
| Gay/Lesbian | 2.21 (0.87) |  | 2.71 (0.86) |  | 1.95 (0.74) |  |
| Something Else | 2.34 (0.86) |  | 2.73 (0.94) |  | 2.23 (0.73) |  |
| Race/Ethnicity |  |  |  |  |  |  |
| White, Non-Hispanic | 1.97 (0.71) | **<0.01** | 2.52 (0.79) | **0.191** | 1.86 (0.66) | **<0.01** |
| Hispanic | 2.18 (0.70) |  | 2.46 (0.76) |  | 2.09 (0.67) |  |
| Black, Non-Hispanic | 1.95 (0.69) |  | 2.31 (0.73) |  | 1.91 (0.70) |  |
| Multiracial | 1.94 (0.67) |  | 2.51 (0.77) |  | 1.78 (0.63) |  |
| Other, single race | 2.23 (0.66) |  | 2.38 (0.87) |  | 1.96 (0.74) |  |

1. Although the p-value for the ANOVA was significant, the Tukey adjusted post-hoc tests showed no significant differences between groups.
